# Large band gap quantum spin Hall insulators in plumbene monolayer decorated with amidogen, hydroxyl and thiol functional groups

**DOI:** 10.1039/d2na00912a

**Published:** 2023-05-22

**Authors:** Sumaiya Jahan Tabassum, Tanshia Tahreen Tanisha, Nishat Tasnim Hiramony, Samia Subrina

**Affiliations:** a Department of Electrical and Electronic Engineering, Bangladesh University of Engineering and Technology Dhaka 1205 Bangladesh samiasubrina@eee.buet.ac.bd ssubr002@ucr.edu +88-02-9668054 +880-19-3795-9083 +88-02-9668054

## Abstract

Two-dimensional Quantum Spin Hall (QSH) insulators featuring edge states that are topologically protected against back-scattering are arising as a novel state of quantum matter. One of the major obstacles to finding QSH insulators operable at room temperature is the insufficiency of suitable materials demonstrating the QSH effect with a large bulk band gap. Plumbene, the latest group-IV graphene analogous material, shows a large SOC-induced band gap opening but the coupling between topological states at different momentum points makes it a topologically trivial insulator. Pristine plumbene can be chemically functionalized to transform it from a conventional insulator to a topologically non-trivial insulator with a considerable bulk band gap. In this work, three new QSH phases in plumbene have been theoretically predicted through functionalization with amidogen (–NH_2_), hydroxyl (–OH) and thiol (–SH) groups. The derived electronic properties show non-trivial topological states in plumbene with very high bulk band gaps ranging from 1.0911 eV to as high as 1.1515 eV. External strain can be used to further enhance and tune these bulk gaps, as demonstrated in this work. We also propose a H-terminated SiC (0001) surface as a suitable substrate for the practical implementation of these monolayers to minimize lattice mismatch and maintain their topological order. The robustness of these QSH insulators against strain and substrate effects and the large bulk gaps provide a promising platform for potential applications of future low dissipation nanoelectronic devices and spintronic devices at room temperature.

## Abstract 2

Three proposed quantum spin Hall insulators based on chemically functionalized plumbene monolayer are found to possess giant bulk band gaps, which are strain-tunable. Topological non-triviality remains intact even upon introduction of a substrate.

## Graphical abstract

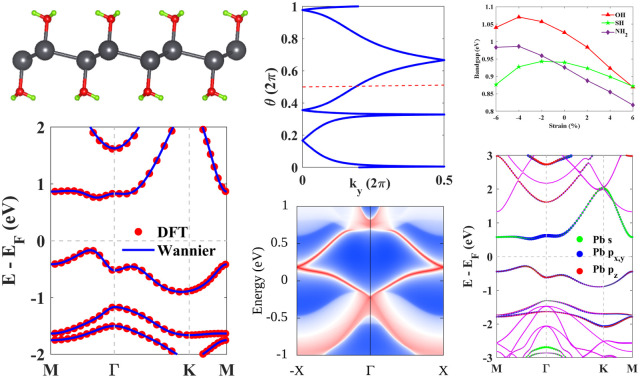

## Introduction

Topological insulators are a novel class of materials that display bulk band gaps but have gapless edge or surface states that are protected against back-scattering from non-magnetic impurities because their directionality and spin are coupled.^1^ These unique states can result in dissipation-less transport and can be used in low power electronic applications as well as topological quantum computing.^2^ Kane and Mele were the first to predict a quantum spin Hall (QSH) phase in graphene in 2005.^3^ Although graphene was the first material predicted to show the QSH phase, an experimentally proven QSH phase was first found in mercury-telluride (HgTe) quantum wells^4^ and InAs/GaSb quantum wells.^5^ All well-known topological materials feature band inversion, *i.e.*, the conventional orderings of their conduction bands and valence bands at some high-symmetry momentum points are reversed.^6^

Group-IV two-dimensional materials possessing a honeycomb lattice structure such as graphene, silicene, germanene, stanene and plumbene have been a topic of intensive research since the discovery of graphene in 2004.^7^ The linear dispersion of energy near the Fermi level in these materials has led to very high carrier mobilities where electrons can act as massless Dirac fermions.^8,9^ As mentioned earlier, the QSH phase in graphene has been found through theoretical approaches; however, the SOC in graphene is very weak and the SOC induced band gap opening at the degenerate Dirac point is in the order of 10^−5^ eV.^10,11^ This implies that graphene can only be realized as a QSH insulator at unrealistically low temperatures. Other two-dimensional graphene analogous materials such as silicene, germanene and stanene have also been reported to be topological insulators.^12–14^ It has been predicted that these novel 2D materials will support the QSH effect with a SOC band gap opening of 1.55 meV for silicene, 23.9 meV for germanene and 0.3 eV for stanene. As we go to higher atomic number atoms, we see a rise in the SOC-induced band gap opening because the magnetic field due to orbital motion increases with a higher atomic number, and hence, the coupling effect between the orbital magnetic field and spin magnetic moment also increases. Although silicene, germanene and stanene show higher band gaps in the presence of SOC than graphene, it is difficult to find use of these materials as QSH insulators at high temperature. Plumbene, the latest graphene analogous two-dimensional structure, has emerged as a promising candidate for application as a room temperature QSH insulator due to its high band gap opening in the presence of SOC.^15^ However, the difference between plumbene and other group-IV 2D structures is that a topologically trivial property has been anticipated for pristine plumbene.^16,17^ The source of this topologically trivial property of pristine plumbene arrives from the coupling of linear Dirac bands at the K point and quadratic non-Dirac bands at the Γ point as explained in ref. 18. The quadratic non-Dirac bands at the Γ point are special in plumbene and are not seen in the other group-IV monolayers. Both of these states at the Γ point and K point are topologically non-trivial.^18^ However, the coupling between these two results in the global topologically trivial property of plumbene. Even though pristine plumbene is topologically trivial, the strong SOC makes it appealing for application as a QSH insulator at room temperature through some modification.

Certain schemes such as chemical decoration^18–20^ and application of an electric field^22^ have been employed to modify the topological behavior of group IV monolayers. Wang *et al.* have demonstrated that two dimensional arsenene oxide, where oxygen is used as the surface functional group for two-dimensional arsenene, is suitable for application as a room temperature QSH insulator.^21^ The problem with applied electric field is that the band gap opening is very small to be useable at room temperature.^22,23^ In contrast chemical functionalization has opened up large band gaps in low buckled plumbene monolayer as well as bringing in topological behavior that can be applicable at room temperature.^18–20,24,25^ Over the years different types of functional groups have been used to passivate two-dimensional hexagonal structures. The hydrogenation and halogenation of two-dimensional monolayer Xenes (X = C, Si, Ge, Sn, Pb) have been predicted to open up large band gaps,^26,27^ as high as 1.34 eV in the case of fluorinated plumbene monolayer.^19^ Organic functional groups such as methyl (–CH_3_),^25^ ethynyl (–C_2_H)^18^ and CH_2_–O–CH_3_ ^28^ have also shown large band gap QSH phases in plumbene with bulk gaps 0.9818, 0.912 and 0.8 eV. Another type of functional group used is cyanogen (–CN) which gives a band gap of 0.92 eV in plumbene monolayer and the resulting material is a QSH insulator. The way that chemical functionalization changes the topological invariant is by breaking the band degeneracy at the K/K′ point and thus destroying the constructive coupling of the two different momentum points, namely Γ and K.

Despite the opening of giant bulk gaps, not all functional groups can ensure the application as room temperature QSH insulators. Plasma hydrogenation and halogenation carried out experimentally have shown increasing defects and disorders on the substrate.^29^ Moreover, hydrogenated surfaces are prone to becoming oxidized when exposed to the ambient.^30,31^ The preparation of high quality hydrogenated and halogenated lead films remains a challenge due to these problems. Most of the studies done on plumbene monolayer in recent years are limited to the realm of theoretical predictions. Even if some functional groups are experimentally feasible, the environmental impacts of some functional groups such as cyanogen also need to be considered. Hence, the search for groups that are experimentally synthesizable with low disorders and have low toxicity still remains a challenge in the application of plumbene thin film QSH insulators. The amidogen (NH_2_) functional has previously shown promise in the functionalization of nanostructures^32–36^ and amidogen functionalized two-dimensional bismuthene and antimonene films have shown non-trivial Z_2_ invariant and helical edge states.^37^ Double-sided passivation of a graphene-like borophene structure with amidogen has also opened up a large bulk global band gap.^38^ Other functionals such as hydroxyl (–OH) and thiol (–SH) have shown phase transitions to a topological insulator phase in germanene,^39,40^ arsenene^41^ and bismuthene^42^ monolayers with bulk band gaps and helical edge states, but these functionals remain unexplored in plumbene monolayers.

Inspired by the epitaxial growth of plumbene on a nano-watercube,^43^ in this work the effect of amidogen, hydroxyl and thiol radical groups on the QSH phase in plumbene has been studied. We observe non-trivial topological states in plumbene with very high bulk band gaps ranging from 0.926 to as high as 1.0259 eV. We also observe the tunability and robustness of the proposed QSH insulators, as reported by previous investigations on other 2D materials. For instance, a first-principles study of single layer RuClBr demonstrates electronic correlation dependent quantum phase transitions and shows that the QAVHE phase has complete spin and valley polarization.^44^ The electron valley polarization is also susceptible to magnetic control by an external magnetic field. More studies such as that done by Zhang *et al.*^45^ on Nb_2_O_3_ shows the emergence of a nontrivial QAH phase in the material when SOC is considered, which is robust against biaxial strain and gives a tunable band gap. This motivated us to examine the robustness and tunability of the non-trivial topological phase by applying biaxial strain. Furthermore, H-terminated SiC (0001) has been proposed as an ideal substrate for maintaining their topological order in the experimental realization of these materials.

## Computational details

All the density functional theory (DFT)^46,47^ based first-principles calculations in this work were performed using Quantum ESPRESSO.^48–51^ The plane-wave basis set and the projector augmented-wave (PAW) method^52^ as implemented in Quantum ESPRESSO have been employed. To describe the exchange-correlation energy, the generalized gradient approximation (GGA) within the Perdew–Burke–Ernzerhof (PBE) functional^53^ has been adopted. A Monkhorst–Pack mesh^54^ has been used here to sample the Brillouin zone. A value of 60 Ry has been used as the kinetic energy cutoff for the wave function and 600 Ry has been set as the kinetic energy cutoff for charge density and potential. For geometry optimization, the atoms and cell parameters are relaxed until the total energy changed less than 7.3 × 10^−8^ Ry between two consecutive scf steps and all components of all forces were smaller than 3.9 × 10^−4^ Ry bohr^−1^. Since the PBE functional is known to underestimate the band gap, the Heyd–Scuseria–Ernzerhof (HSE06)^55^ functional has also been employed to get the corrected band gap values. The HSE band gap has been calculated from the difference of the lowest unoccupied and highest occupied levels determined by self-consistent calculations. A gamma centered *k* point grid size of 10 × 10 × 1 was employed to sample the Brillouin Zone. The *q* sampling of the Fock operator has to be a factor of the *k* mesh, and therefore, the *q* mesh size was chosen to be 5 × 5 × 1. Spin–orbit coupling has been included in all of the calculations. Using the following equation, we determine the formation energy per atom for the three structures.*E*_f_ = *E*(PbR) − *E*(Pb) − *E*(R)Here, *E*(PbR), *E*(Pb) and *E*(R) are the energies of the chemically decorated monolayer per Pb atom, of the pristine monolayer per Pb atom and of the R functional group respectively. Here, R = NH_2_, OH and SH.

To confirm the presence of non-trivial topological order in the functionalized monolayers, maximally localized Wannier functions (MLWF) are calculated and an MLWF tight-binding Hamiltonian is constructed using the tool Wannier90.^56^ Then the ℤ_2_ invariants are calculated with the help of WannierTools.^57^ In addition, the edge state spectrum is calculated for each monolayer based on the iterative Green's function method^58^ as implemented in WannierTools.

Then, the evolution of the band gaps of the functionalized monolayers with the application of external biaxial strain has been studied since strain engineering has previously been demonstrated as an excellent method to tune material properties. The lattice constant is changed according to the following equation to observe the effect of external biaxial strain.
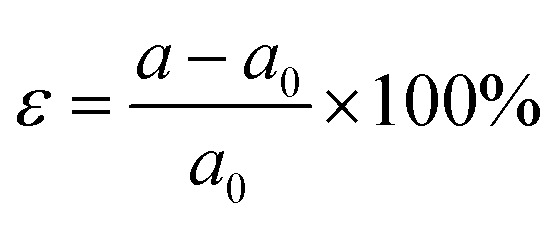
Here, *a* is the lattice constant under strain and *a*_0_ is the lattice constant of the fully relaxed, unstrained structure. We applied biaxial strain ranging from −6% to +6% on the decorated monolayers.

## Results and discussion

**Fig. 1:**
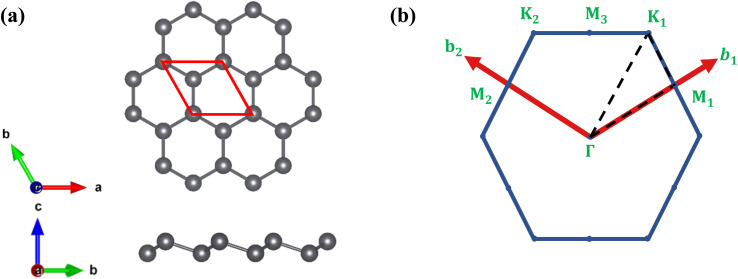
(a) The top-view (top) and side-view (bottom) of the geometry-optimized structure of plumbene and (b) Brillouin zone corresponding to the honeycomb lattice with the high-symmetry points labelled. The unit cell is marked in the top-view with a red parallelogram.

Plumbene has a two-dimensional hexagonal lattice similar to graphene and it has three possible structures: planar, low-buckled and high-buckled.^18,24,59^ Pb atoms are sp^3^ hybridized in buckled plumbene, whereas they are sp^2^ hybridized in planar plumbene. Since sp^2^ bonding between Pb atoms is unfavourable, geometries with high and low buckling are more stable than those with a planar geometry.^15^ Among the two buckled configurations, the high-buckled one is more stable in pristine plumbene but the low-buckled one has been reported to be more stable when plumbene was decorated with some functional groups.^24^ For this reason, low-buckled plumbene, in pristine and decorated forms has been studied in this work. Fig. 1 shows the schematic structure of plumbene and the Brillouin zone of the hexagonal crystal lattice.

**Fig. 2:**
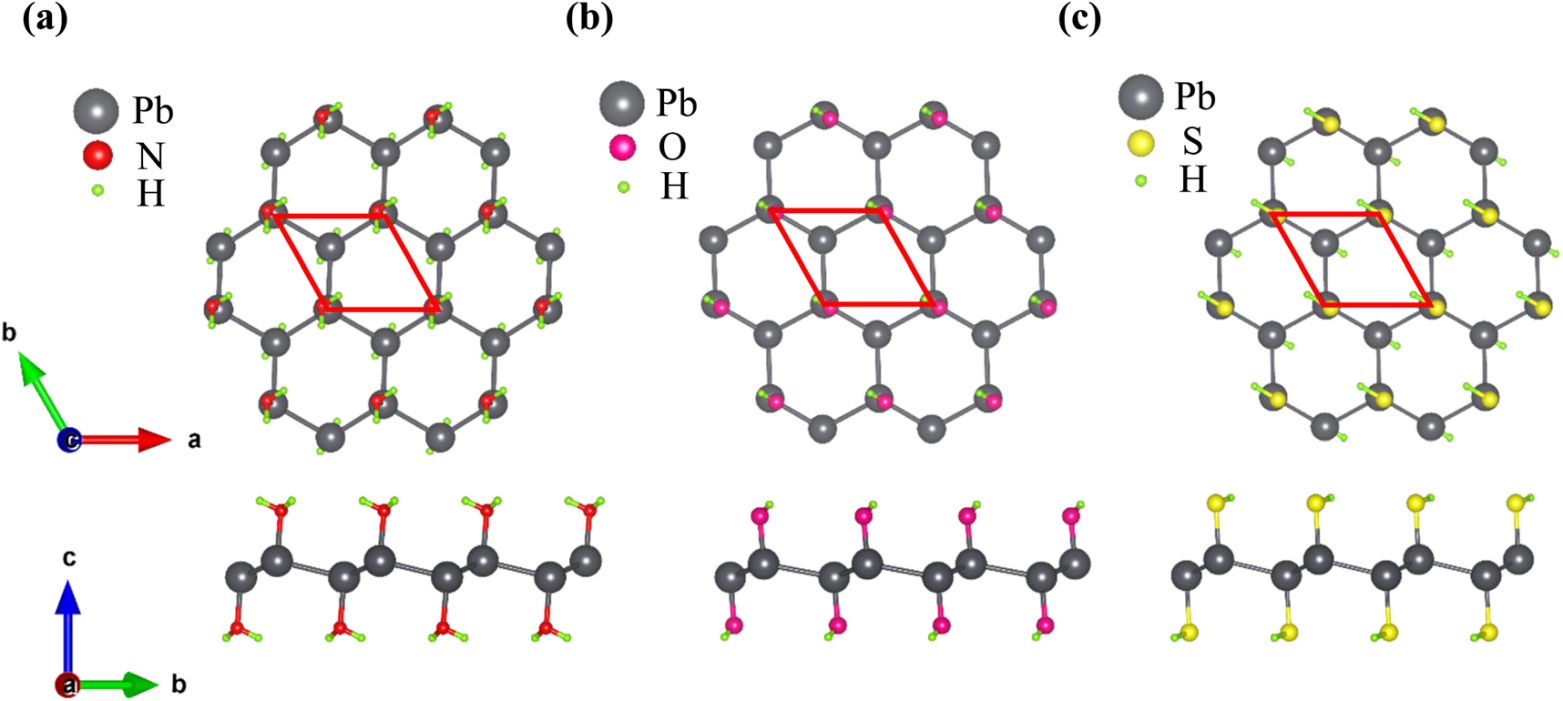
The top-view (top) and side-view (bottom) of the geometry-optimized structures of (a) PbNH_2_, (b) PbOH and (c) PbSH. The unit cell is marked in the top-view of each structure with a red parallelogram.

The schematic structures of the functionalized monolayers are shown in Fig. 2. The unit cell of each functionalized monolayer consists of two Pb atoms, each of which is bonded to a functional group (–NH_2_ or –OH or –SH). The optimized lattice parameters (a), Pb–Pb bond lengths (*l*) and buckling heights (*d*) of the pristine and three functionalized plumbene monolayers have been listed in Table 1.

**Table 1:** The crystal parameters of the unit cell in relaxed structures including the lattice constant (a), Pb–Pb bond length (*l*), bucking height (*h*), band gap calculated with GGA (*E*_g_), band gap with GGA + SOC (
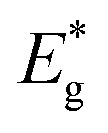
), band gap with HSE + SOC (*E*_g,HSE_) and formation energy (*E*_f_)

Structures	*a* (Å)	*l* (Å)	*h* (Å)	*E*_g_ (eV)	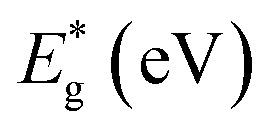	*E*_g,HSE_ (eV)	*E*_f_ (eV)
Pb	4.9241	2.9971	0.9489	0	0.4827	—	—
PbNH_2_	5.1555	3.0625	0.7119	0.4605	0.926	1.1359	−2.6407
PbOH	5.2865	3.1152	0.5920	0.458	1.0259	1.1515	−3.3711
PbSH	5.1991	3.0993	0.7311	0.540	0.9403	1.0911	−2.3555

The lattice constants and the Pb–Pb bond lengths are greater in the functionalized monolayers in comparison to those in pristine plumbene. On the other hand, the buckling height has dropped upon functionalization due to the weak hybridization between the σ and π orbitals.^19^ Similar results have been obtained in other functionalized plumbene structures.^19,25^ The lowering of the buckling height plays a vital role in the determination of the electronic properties of these materials. The formation energy of each functionalized structure is calculated since it is an indicator of stability of the structure. The obtained values of formation energy are given in Table 1. All of these values are negative, which shows that there exists no phase separation between the Pb atom and the functional groups and also ensures electronic stability of these structures.

**Fig. 3:**
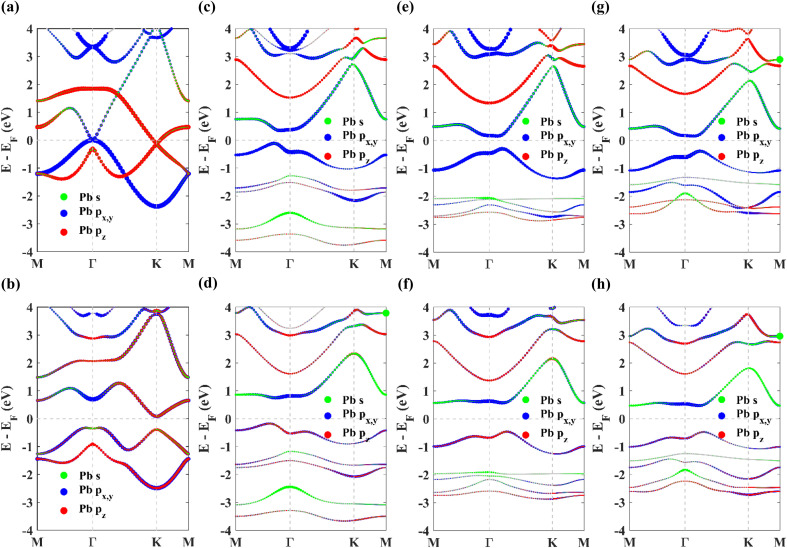
The orbital-resolved band structures of (a) pristine plumbene without SOC, (b) pristine plumbene with SOC, (c) PbNH_2_ without SOC, (d) PbNH_2_ with SOC, (e) PbOH without SOC, (f) PbOH with SOC, (g) PbSH without SOC, and (h) PbSH with SOC.

Next, to explore the topological non-triviality of the materials, their electronic band structures are calculated. The band structures of pristine plumbene monolayer are shown in Fig. 3(a) and (b) without and with SOC respectively. Without SOC, the band structure does not feature a gap. Upon inclusion of SOC, it can be seen that an indirect band gap opens up. The conduction band minimum (CBM) lies at K, whereas the valence band maximum (VBM) lies close to Γ. SOC opens up a significant band gap of 0.413 eV. This value is in good agreement with reported values of 0.421 eV ^19^ and 0.44 eV.^18^ Without SOC, in the band structure of pristine plumbene, two degeneracies exist simultaneously. There is a quadratic band dispersion with degeneracy at Γ and a linear Dirac band dispersion involving degeneracy at the K point of the Brillouin zone. It is evident from the orbital projections that the degenerate bands at the K point are dominated by p_*z*_ orbitals. The coupling between the two topological states around the Γ and K/K′ points causes pristine plumbene to be a global topologically trivial insulator.^18^

The band structures of the chemically decorated plumbene monolayers have been shown in Fig. 3(c)–(h). In the absence of SOC (Fig. 3(c), (e) and (g)), the band gap at the K point is significantly enhanced in the functionalized monolayers in comparison with that of pristine plumbene monolayer (Fig. 3(a)). The conduction band minima (CBM) and valence band maxima (VBM) of all the band structures are located close to the Γ point. Through the projection of the bands onto different atomic orbitals, it can be observed that the p_*z*_-rich bands which were observed at the K point in pristine plumbene have moved apart from each other and away from the Fermi energy level in decorated plumbene. Functionalization leads to lifting of degeneracy in the band structure at the K point which paves the way to the realization of a non-trivial topological phase in decorated plumbene monolayers. The effect of SOC on the band structures is significant. Once the presence of SOC is taken into account (Fig. 3(d), (f) and (h)), the conduction bands are lifted upwards, whereas the valence bands are pulled downwards, producing larger global indirect gaps. The band gaps calculated using the PBE functional for the monolayers are: 0.926 eV in PbNH_2_, 1.0259 eV in PbOH and 0.9403 eV in PbSH, as tabulated in Table 1. These band gaps are ample for using these materials at room temperature. Using HSE, the band gaps of the materials are further enhanced. The calculated HSE band gaps for the three decorated monolayers are: 1.1359 eV in PbNH_2_, 1.1515 eV in PbOH and 1.0911 eV in PbSH. Compared to the PBE band gaps, these HSE band gaps are considerably higher, as expected. From the projected band structures, it can be seen that an s–p_*x*,*y*_ band inversion exists both in the absence and presence of SOC. This band inversion is indicative of the existence of non-trivial band topology in chemically decorated plumbene, whereas SOC is only responsible for opening up a giant band gap in the band structures.^19^ –NH_2_, –OH and –SH functionalized plumbene monolayers possess giant band gaps (>0.9 eV) as observed in Fig. 3(d), (f) and (h). This makes them potential candidates for room temperature applications.

**Fig. 4:**
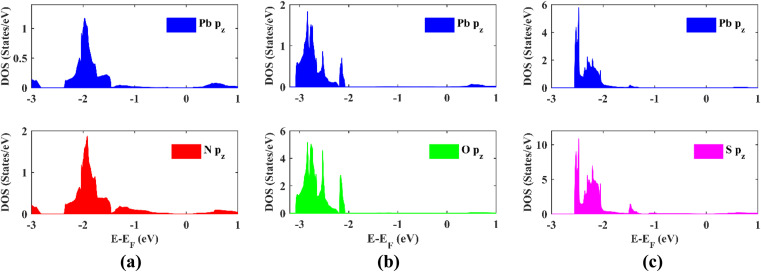
The projected density of states (PDOS) of the p_*z*_ orbitals of (a) Pb and N atoms in PbNH_2_, (b) Pb and O atoms in PbOH, and (c) Pb and S atoms in PbSH in the absence of SOC.

The projected density of states (PDOS) of the p_*z*_ orbital of Pb atoms and p_*z*_ orbital of N, O and S atoms in PbNH_2_, PbOH and PbSH monolayers respectively has been shown in Fig. 4 without taking SOC into consideration. The contribution of the p_*z*_ orbitals mostly exists far away from the Fermi energy. This is consistent with the projected band structures where it was observed that chemical decoration causes the contributions of p_*z*_ orbitals to move away from the Fermi energy level. This eliminates the degeneracy existing at the K point of the Brillouin zone in the band structure of pristine plumbene as mentioned earlier. Furthermore, it is apparent from Fig. 4(a) that the DOS pattern of p_*z*_ orbitals of Pb atoms and that of N atoms in PbNH_2_ monolayer align with one another, hinting towards the formation of covalent bonds between these orbitals. This argument is also valid for Pb and O atoms in PbOH monolayer in Fig. 4(b) and for Pb and S atoms in PbSH monolayer in Fig. 4(c). The bond formation is the reason why the contribution of the p_*z*_ orbitals of Pb atoms moves away from the Fermi level.

**Fig. 5:**
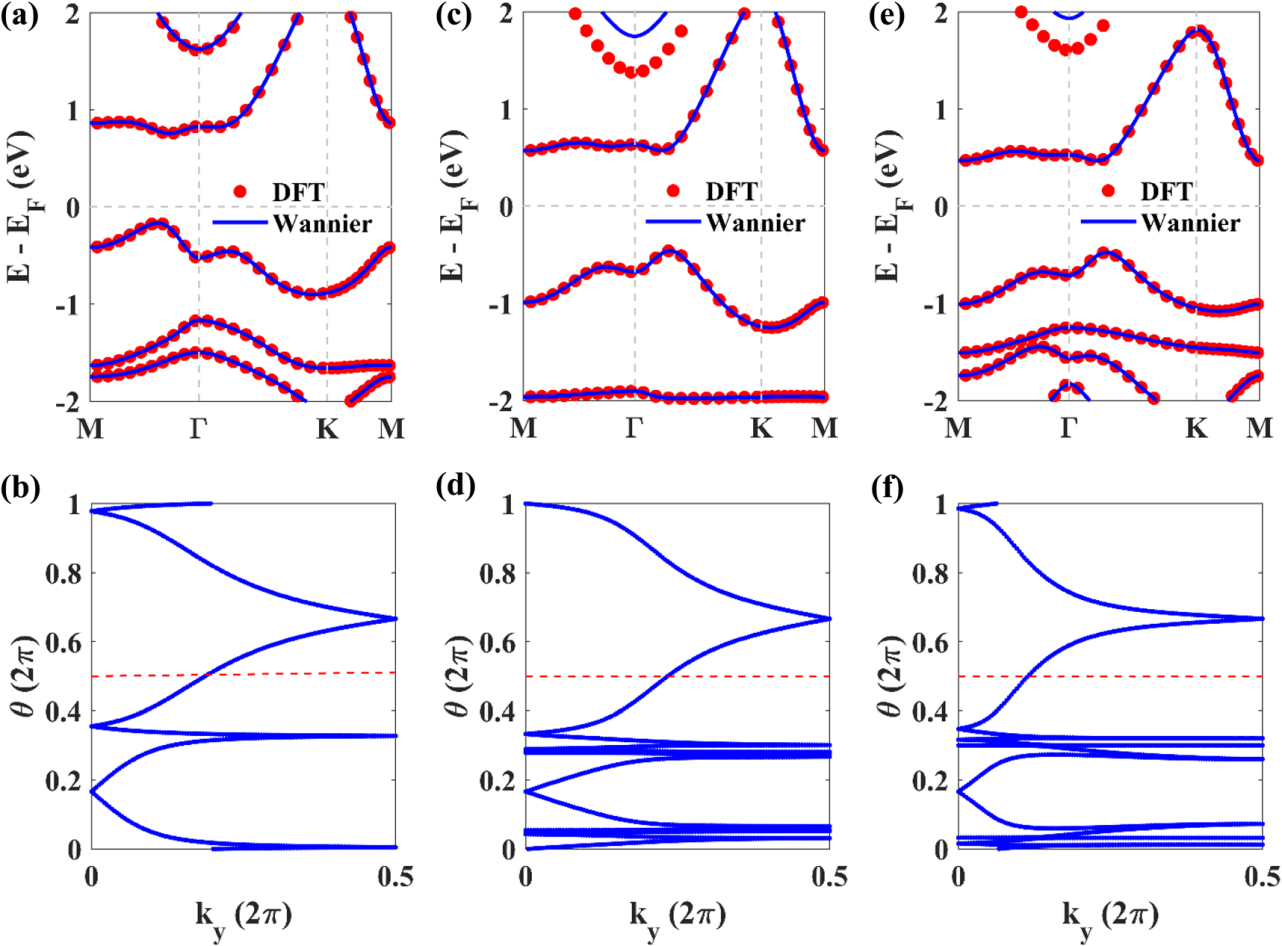
Band structures calculated by using DFT and Wannier functions shown in the same plot for (a) PbNH_2_, (c) PbOH and (e) PbSH; evolution of the Wannier charge centers (WCCs) of (b) PbNH_2_, (d) PbOH and (f) PbSH.

To confirm the topological non-triviality of the functionalized plumbene monolayers, maximally localized Wannier functions (MLWFs) are obtained and a tight-binding Hamiltonian is constructed on the basis of these functions. The results obtained for PbNH_2_, PbOH and PbSH are shown in Fig. 5. The fact that the Wannier interpolated band structure coincides with the electronic band structure obtained from DFT (Fig. 5(a), (c) and (e)) asserts the correctness of Wannierization for each monolayer. The Wannier charge center (WCC) curves are obtained from the Wannier functions. From the WCC curves, ℤ_2_ can be computed by counting the number of times an arbitrary horizontal line crosses the WCC curves. If the number of intersections is even, ℤ_2_ is 0, indicating that the material is topologically trivial whereas for an odd number of intersections, ℤ_2_ is 1, implying that the material is topologically non-trivial. The WCC curves of –NH_2_, –OH and –SH decorated plumbene have been shown in Fig. 5(b), (d) and (e) respectively. An odd number of crossings can be observed between the red horizontal reference line and the WCC curves, indicating that ℤ_2_ is 1.

**Fig. 6:**
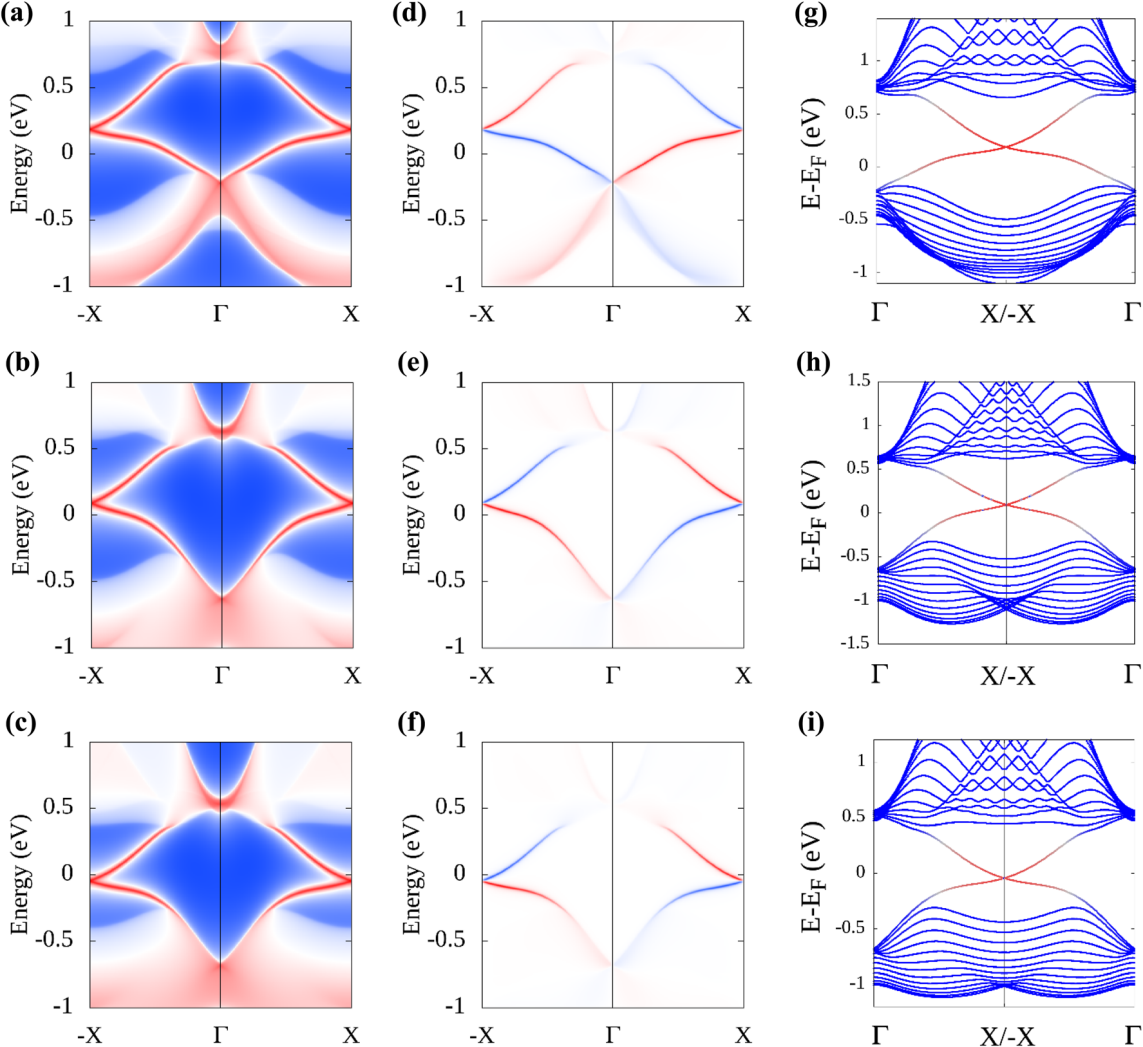
Calculated total and spin edge density of states of PbNH_2_ are shown in (a) and (d) respectively, and those for PbOH and PbSH are shown in (b), (e) and (c), (f) respectively. The calculated band structure of a zigzag-edged nanoribbon of PbNH_2_ is shown in (g), and those for PbOH and PbSH are shown in (h) and (i) respectively.

**Fig. 7:**
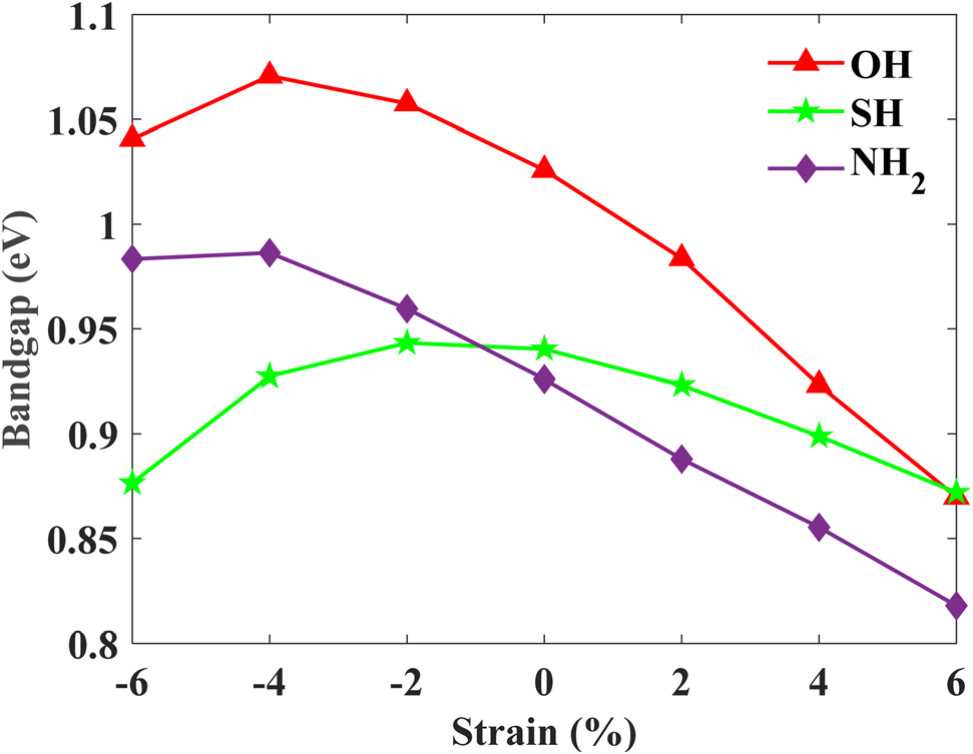
Calculated band gaps (with SOC) as a function of external biaxial strain.

Additionally, the edge state spectrum is calculated for each monolayer. 2D topological insulators have a band gap in the bulk and gapless topologically protected edge states at the edges. Thus, while the bulk is insulating, the edges conduct. In pristine plumbene, the breakdown of the QSH effect is attributed to the unavoidable interaction between the two pairs of the topologically protected edge states.^18^ If there are odd number of edge states, the QSH state would survive in the system. The gapless edge states in PbNH_2_ are shown in Fig. 6(a). The edge states connect the conduction band and the valence band by crossing in a linear fashion, resulting in a zero band gap. The spin-projected edge states have been displayed in Fig. 6(d). The red and blue colours correspond to the projections of spin-up and spin-down electrons respectively. It is apparent that the edge states are counter propagating as one would expect. The calculated bands of a zigzag nanoribbon of PbNH_2_ can be seen in Fig. 6(g), where the gapless edge states are visible. The corresponding figures for PbOH and PbSH are shown in Fig. 6(b), (e), (h) and (c), (f), (i) respectively. Next, the robustness and tunability of the band gap of the said structures are investigated. Strain engineering has reported to yield fascinating properties in two-dimensional materials. Practically, materials can be strained through different schemes, such as, lattice mismatch,^60,61^ flexible substrates,^62,63^ piezoelectric substrates,^64^ patterned substrates,^65^ AFM tips,^66–68^*etc.*Fig. 7 depicts how the bulk band gap changes with external strain. The trend of change in the band gap with external strain is similar in all three materials. For all of the decorated monolayers, the global band gap keeps increasing with compressive strain and reaches a maximum value, and then keeps decreasing with further compressive strain. The global band gaps keep increasing for biaxial compressive strain with a maximum band gap of 0.9863, 1.0707 and 0.9433 eV for PbNH_2_, PbOH and PbSH at −4%, −4% and −2% strain respectively. The band gaps show a continually decreasing trend in the case of tensile biaxial strain. It is evident that applying strain can help tune the band gap of the materials, which is very advantageous in many applications. On the other hand, higher band gaps achieved by applying compressive strain can help make use of the QSH phase in these materials at higher temperatures.

**Fig. 8:**
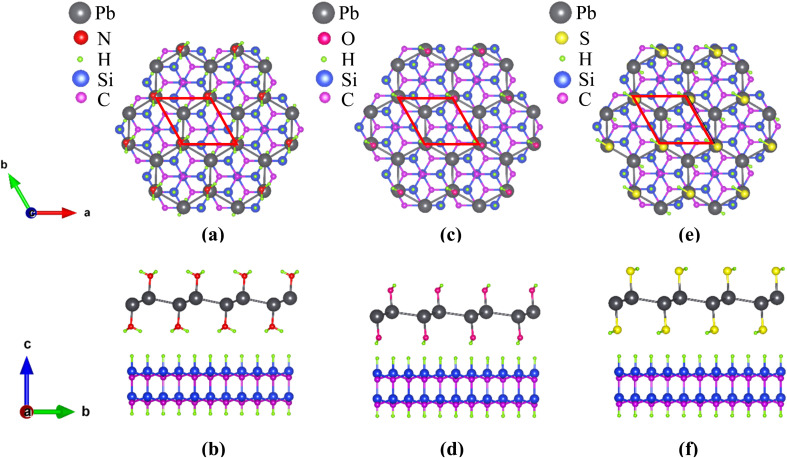
The top-view and side-view of PbNH_2_ standing on a 
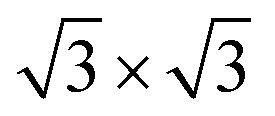
 supercell of the SiC substrate are shown in (a) and (b) respectively. The unit cell of the system is in a commensurate (
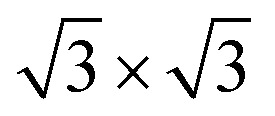
) *R*30° reconstruction of SiC (0001). The corresponding figures for composite structures based on PbOH and PbSH are shown in (c) and (d) and (e) and (f) respectively.

Now, for practical applications, free-standing two-dimensional films must be deposited or grown on a substrate by different deposition or epitaxial processes. In 2019, Yuhara *et al.* reported the epitaxial growth of plumbene on a Pd_1−*x*_Pb_*x*_ (111) alloy surface.^43^ To investigate the potential for experimental realization of decorated monolayers studied in this work, we deposit them on a H-terminated (
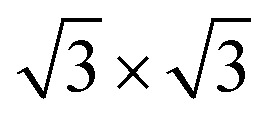
) *R*30° reconstruction of the SiC (0001) surface. The substrate consists of a SiC bilayer with H atoms on both the top and the bottom surface. The top-view and side-view of the relaxed structure of PbNH_2_ standing on the substrate are illustrated in Fig. 8(a) and (b) respectively. The structures corresponding to PbOH and PbSH are shown in (c) and (d) and (e) and (f) respectively.

**Fig. 9:**
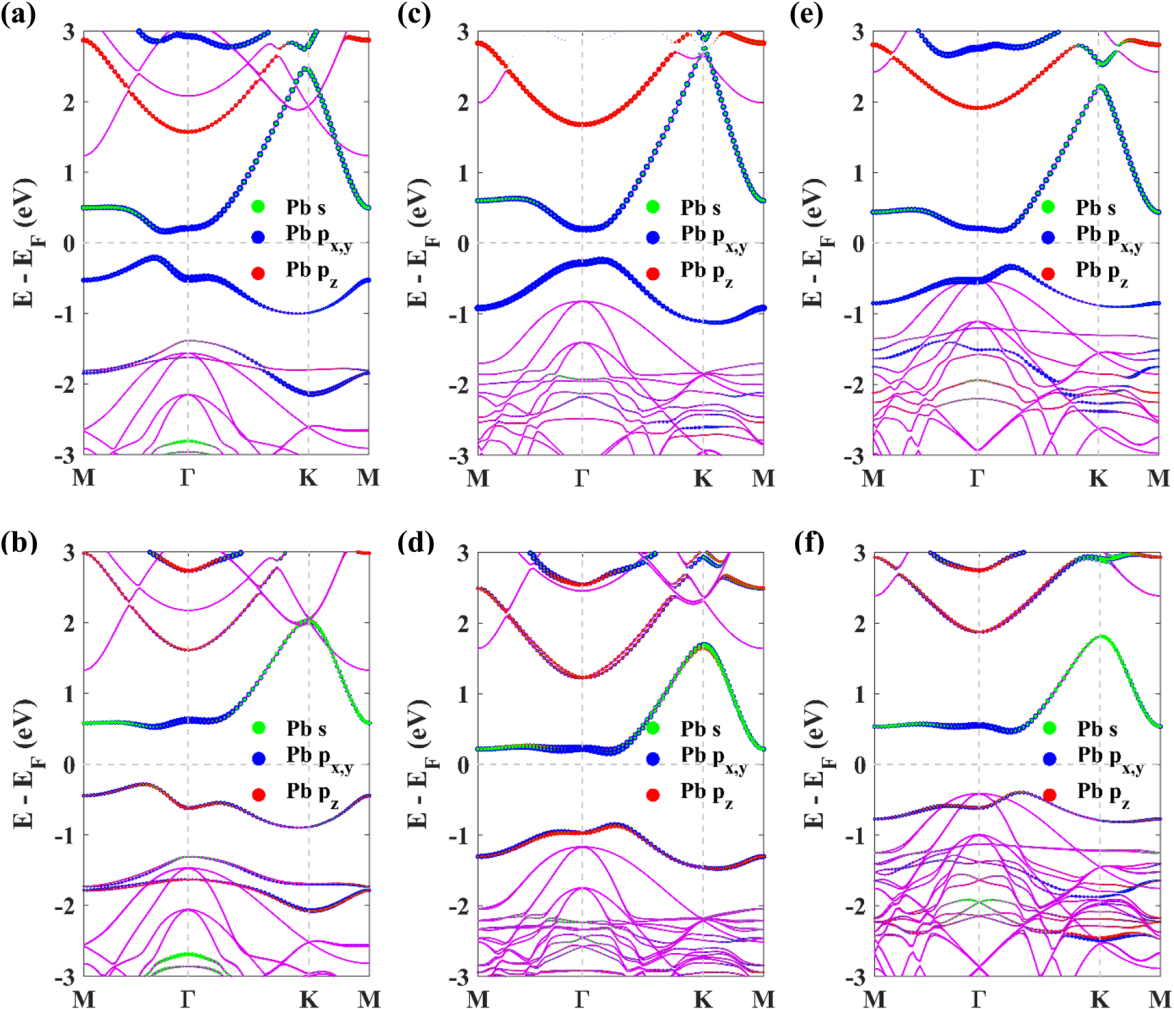
The orbital-resolved band structures of the composite structure corresponding to PbNH_2_ without and with SOC are depicted in (a) and (b) respectively, and those for PbOH and PbSH are shown in (c) and (d) and (e) and (f) respectively.

The obtained band structures of the composite structures without and with SOC are shown in Fig. 9. The structure is modeled by placing the SiC bilayer under the decorated monolayers where the bottom H atoms of SiC are kept fixed in position. The unit cell of the composite structure consists of 26 atoms for PbNH_2_ and 24 atoms for PbOH and PbSH. To correctly account for the van der Waals interaction, a dispersion corrected DFT method (optB88-vdW) is used.^69,70^ The lattice mismatch between the PbNH_2_ layer and SiC substrate is 4.468% and that for the PbOH and PbSH layers is 1.879 and 3.542% respectively. The distances between adjacent layers are 2.4668 Å, 1.4824 Å and 2.2360 Å respectively for PbNH_2_, PbOH and PbSH. The interlayer distances are greater than the H–H bond length, which indicates the presence of van der Waals interactions in the interface. The projected band structures show that the bands near the Fermi level are unaffected by the substrate. The substrate bands do not affect the band inversion, and hence, the topological order of the monolayers. However, the global band gaps are slightly changed after the growth of the substrates on the monolayers. Table 2 lists the band gaps without and with SOC when the substrate is brought into the picture.

**Table 2:** Interlayer distances, lattice mismatches and global band gaps (without and with SOC) of the decorated monolayers on the SiC substrate

Material	Lattice mismatch (%)	Interlayer distance (Å)	*E*_g_ without SOC (eV)	*E*_g_ with SOC (eV)
PbNH_2_ on SiC	4.468	2.4668	0.382	0.811
PbOH on SiC	1.879	1.4824	0.438	1.010
PbSH on SiC	3.542	2.2360	0.511	0.866

## Conclusion

In this work, three quantum spin Hall insulators based on plumbene monolayer have been theoretically predicted and analyzed. The negative formation energy reveals that they possess structural stability. The electronic band structure, ℤ_2_ invariant and gap-connecting, counter-propagating edge states in these monolayers bear signatures of their topological non-triviality. The band structure reveals that they possess a bulk gap and the edge density of states reveals that there are gap-connecting states at the edges. The giant bulk band gap, tunable by external biaxial strain, ensures that they can be used at room temperature. It has also been shown here that their topological non-triviality is robust against strain, which makes them useable for practical applications where strain might be inevitable. Biaxial compressive strain creates an even larger band gap and biaxial tensile strain results in a reduced band gap. Furthermore, the monolayers have been placed on a H-terminated SiC (0001) substrate and their band structures obtained thereafter reflect the retention of the quantum spin Hall state, pointing to the possibility of practical realization and thus adding one more point to their applicability in practical scenarios. These edge states of the quantum spin Hall insulators are protected by time-reversal symmetry. The robust gapless edge states protect these chemically decorated monolayers from back-scattering,^71^ which makes them prospective candidates for use in dissipation-less transport devices and low-power quantum electronic devices at room temperature.

## Conflicts of interest

There are no conflicts of interest to declare.

## Supplementary Material
